# Evaluation of Hot Pressing Processing by Physical Properties of Ecofriendly Composites Reinforced by Eucalyptus Sawdust and Chamotte Residues

**DOI:** 10.3390/polym15081931

**Published:** 2023-04-19

**Authors:** Juvenil Nunes de Oliveira Júnior, Felipe Perissé Duarte Lopes, Noan Tonini Simonassi, Michel Picanço Oliveira, Fabricio Gomes Gonçalves, Carlos Maurício Fontes Vieira

**Affiliations:** 1 Advanced Materials Laboratory, State University of the North of Rio de Janeiro Darcy Ribeiro—UENF, Av Alberto Lamego, 2000, Campos dos Goytacazes 28013-602, RJ, Brazilvieira@uenf.br (C.M.F.V.); 2 Department of Forest and Wood Sciences, Federal University of Espírito Santos—UFES, Av. Governador Lindemberg, 316, Jerônimo Monteiro 29550-000, ES, Brazil

**Keywords:** biocomposites, vegetal resin, physical properties, chamotte, eucalyptus sawdust, particleboards

## Abstract

The particleboard industry consumes large amounts of raw material, and this type of product consumption has been increasing over the last few years. The research for alternative raw materials becomes interesting, since most of the resources come from planted forests. In addition, the investigation of new raw materials must take into account environmentally correct solutions, such as the use of alternative natural fibers, use of agro-industrial residues, and resins of vegetable origin. The objective of this study was to evaluate the physical properties of panels manufactured by hot pressing using eucalyptus sawdust, chamotte, and polyurethane resin based on castor oil as raw materials. Eight formulations were designed with variations of 0, 5, 10, and 15% of chamotte, and two variations of resin with 10% and 15% of volumetric fraction. Tests of gravimetric density, X-ray densitometry, moisture content, water absorption, thickness swelling, and scanning electron microscopy were carried out. Through the results it can be noticed that the incorporation of chamotte in the manufacture of the panels increased the water absorption and the swelling in thickness, around 100% and the use of 15% of resin decreased, more than 50%, the values of these properties. X-ray densitometry analyzes showed that the addition of chamotte alters the density profile of the panel. In addition, the panels manufactured with 15% resin were classified as P7, the most demanding type on EN 312:2010 standard.

## 1. Introduction

The main source of raw material for the particleboard industry comes from planted forests that basically cultivate Pinus and Eucalyptus species. The world production of wood panels (particleboard) was 104 million m^3^ in 2021 [1], with Brazil responsible for 3.6 million m^3^. Given this, it becomes interesting to investigate other sources of raw material to produce particleboard.

Agro-industrial residues have the potential to be used as raw material for energy production, [2,3,4,5,6,7]. In general, new research with unknown lignocellulosic natural fibers is continuing discovering their potential to be used in engineering materials [8,9,10,11,12].

The manufacture of panels using agricultural residues is an ecologically correct alternative for the technological development in the panel sector, thus providing new sources of raw material.

A waste example is that from ceramic industries, generated in the post-fire brick breaking process, which, in most cases, is improperly disposed of in the environment. The northern region of the state of Rio de Janeiro, in Brazil, has a red ceramic production pole located in the municipality of Campos dos Goytacazes. This leads to the disposal of large amounts of red ceramic pieces, especially after the firing process. Known as chamotte, pieces with inferior quality due in part to failures and inadequacies in the production process, and they are discarded as waste [13,14]. Particles of chamotte can occupy the interstices and voids in particleboard and filling spaces, improving the strengths in some properties and giving a better destination for this waste from the red ceramic industry [8].

In the literature, there are some studies developed with the use of eucalyptus in the production of particleboards, but they have urea-formaldehyde and phenol-formaldehyde as resins [15,16,17,18].

Silva et al. [19] cited that urea-formaldehyde resin is one of the most common resins in the production of particleboards, largely due to its low cost and good mechanical properties. However, the free formaldehyde release during the panel manufacturing process and during its useful life are some of the problems with the use of this type of resin [20].

Alternatively, polyurethane resin based on castor oil has a polyol of vegetable origin with good mechanical properties when used in panels and allows the use of wood particles with higher moisture content when compared to urea-formaldehyde resin [19,21].

Thus, this study has the objective of manufacturing hot-pressed panels using eucalyptus wood residues and residues from the red ceramic industry as raw material, bonded with polyurethane resin based on castor oil, and to evaluate its physical properties. The idea to manufacture by a hot route is to simulate the industry way to produce these types of panels instead of cold processing. The physical properties resulting from cold processing were explored by [12].

## 2. Materials and Methods

This section will be split into raw materials, panels manufacture processing, and tests performed; these topics are described below. 

### 2.1. Raw Materials

The eucalyptus residue was collected (the species is *E. Europhyla*), sieved, and dried in an oven with forced ventilation until it reached constant mass. In the sieving step, a 10-mesh sieve (2 mm opening) was used, and for drying, an oven with circulation and air renewal SL-102/480, Solab brand, was used at 80 °C for 24 h.

Another raw material used was the waste from red ceramic industry. This waste was collected in the form of large pieces of red ceramic blocks, and the powder resulting from the grinding process is called chamotte. The chamotte was dried in an oven at 110 °C for 24 h, comminuted in a Gardelin ball mill for eight hours, and then sieved to 270 mesh (53 µm).

Polyurethane resin based on castor oil was used as the matrix for the panels, produced by the company Imperveg. The ratio of 1:1.8 by mass of polyol derived from castor oil (component B) to the isocyanate groups (component A) was used. Some physical and chemical properties are presented in Table 1.

**Figure 1:**
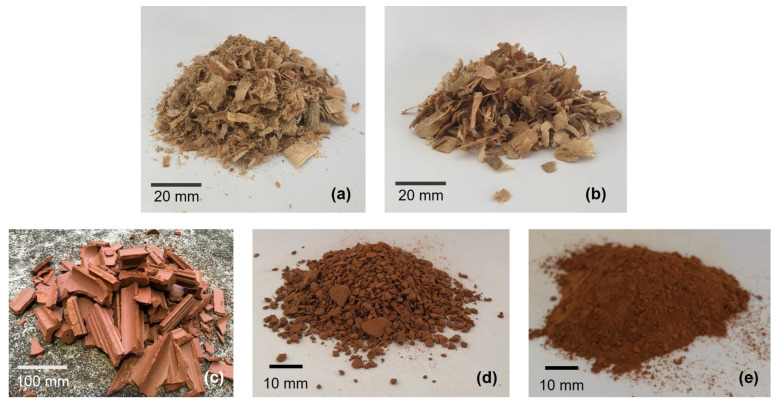
Aspect of raw materials during processing: (**a**) sawdust residue as received, (**b**) retained on sieve of 10 mesh, (**c**) chamote as received, (**d**) chamotte comminuted, and (**e**) chamotte after milling and sieving in 270 mesh.

Figure 1 shows the aspects during the processing of eucalyptus and red ceramic waste (chamotte).

### 2.2. Panels Manufacture

Table 2 presents the experimental formulations with panels composition parameters that were produced. The pressing parameters was 40 kgf/cm^2^ (≅ 4 MPa). For a period of 10 min at a temperature of 100 °C, these parameters produced satisfactory results in the research by Bispo et al. [22], Sugahara et al. [23], where polyurethane resin based on castor oil was also used.

The panels were produced with dimensions of 420 × 420 × 10 mm and nominal density of 0.70 g/cm^3^.

**Figure 2:**
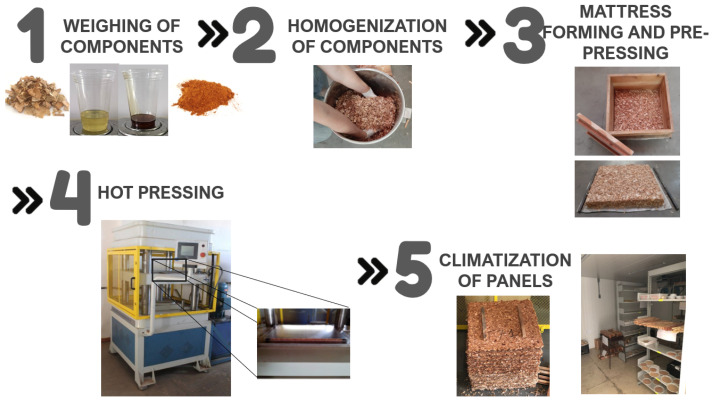
Manufacture panels steps by the hot pressing process.

The processing to manufacture the panels followed the sequence presented in Figure 2. The raw materials were weighed, separately, on a semi-analytical scale according to the experimental design. Following that, they were homogenized using a mortar mixer and then manually for a period of 5 min. The homogenized mixture was placed in a box to carry out the formation of particle bed. The hot pressing was carried out in a hydraulic press with horizontal by flat plates with electric heating, with machine by SOLAB brand and model SL 12, using the parameters previously presented. After the pressing process, the panels were stacked with spacing between them, identified, and sent to the air conditioning room, controlling the temperature inside, that is 30 °C and 40% humidity, where they remained for seven days. Figure 2 shows some stages of panel manufacturing.

### 2.3. Testing Performed

Physical tests carried out on specimens were gravimetric density, X-ray densitometry, moisture content, water absorption, thickness swelling and scanning electron microscopy, JSM manufacturer, and model IT-200.

The physical properties were carried out according to BS EN-323:1993 [24] standard for gravimetric density, BS EN-322:1993 [25] for moisture content, and BS EN-317:1993 [26] for water absorption and thickness swelling after immersion in water. The procedures of these standards are similar to those presented by the ABNT 14810-2 (2018) [27] standard.

The density of particleboard produced by hot pressing is not uniform in the direction of board thickness. The variation in the density of a panel generates a vertical density gradient and can affect the physical and mechanical properties of the panel. For the X-Ray densitometry test, the specimens were cut into dimensions of 50 × 50 mm and then kept in an air-conditioned room until they were analyzed in a GreCon X-Ray densitometer machine, model DAX 6000.

To evaluate the effect of treatments on the physical properties, the analysis of variance (ANOVA) was performed using the “F” test at a level up to 5% of significance after applying the Tukey test. In general, this analysis has the objective of verifying the existence or not of a significant difference between the means of results. In addition, the analysis of the correlation of the properties surveyed through Pearson’s methodology was performed, at 5% error probability. Pearson’s correlation test demonstrates the existing correlation level between the properties, where values close to 1 or −1 represent high correlation (direct or inverse, respectively) and values close to 0 indicate the absence of correlation.

## 3. Results and Discussions

**Figure 3:**
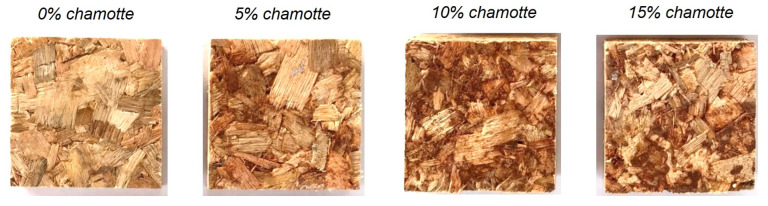
Panels visual comparison with different volume fractions of chamotte.

Figure 3 shows specimens comparing the treatments with different volumetric fractions of chamotte, since the resin variation did not provide any visual variation into panels. It is possible to observe that by increasing the amount of chamotte they are occupying the spaces between eucalyptus sawdust and darkening the specimens; the chamotte is more dark than the eucalyptus, and is close to dark orange.

Table 3 and Table 4 present the results for the physical properties analyzed according to the proposed treatments with 10% and 15% of resin volumetric fraction, respectively.

**Figure 4:**
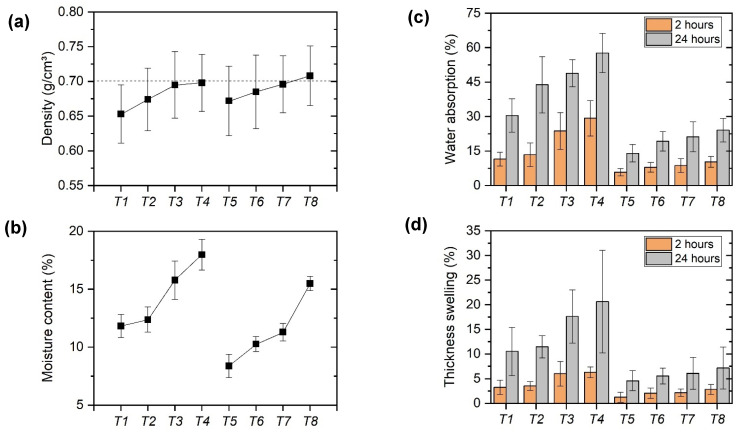
Graphs of physical properties analyzed. (**a**) density, (**b**) moisture content, (**c**) water absorption, and (**d**) thickness swelling.

In Figure 4, the results for the physical properties of panels manufactured by hot pressing are graphically presented for better understanding.

Through Figure 4a it is possible to observe with greater evidence that there is a trend towards an increase in density with the increase in the volumetric fraction of chamotte incorporated in the panel. This behavior was expected, since the chamotte is a ceramic particle with isolated density that is higher than others constituents. In addition, it is noted that only T1 did not have at least one specimen with a nominal density of 0.70 g/cm^3^, according to the standard deviation bars shown in the graph.

Regarding the moisture content, it is noted that the composites manufactured by hot pressing showed higher values than the studies carried out by Buzo et al. [28] and Santos et al. [29]. However, it is observed that the five treatments (T1, T2, T5, T6, T7) proposed in this study meet the minimum requirements described by the ABNT 14810-1 [30] standard, which presents an acceptance range between 5% and 13%. The higher values were likely because of the hygroscopic behavior of ceramic particles. With hot pressing, these particles tend to deposit in the bottom of the mold, and then they are more exposed to ambient as a result.

For water absorption [31], using 20% PU-castor resin we obtained values of 10.5 and 43.4% for panels with macadamia bark and pine wood, respectively, for the 24-h immersion period. Santos et al. [29] produced particle boards with Tauari wood and 16% volumetric fraction of PU-castor, varying the pressing temperature, and obtained 35%, 23%, and 20% of water absorption for the period of 24 h, using temperatures of 90, 110, and 130 ºC, respectively. The general behavior trend follows the same explanation for moisture content.

The thickness swelling decreased with the amount of resin volume fraction and increased with the quantity of chamotte volume fraction. Bertolini et al. [32] obtained thickness swelling values of 4.27 and 4.11% for 2 h and 11.06 and 10.52% for 24 h, using a variation of 12 and 15% PU-castor. Mesquita et al. [33] manufactured panels with açaí fruit fibers using bicomponent polyurethane resin based on castor oil and obtained thickness swelling of 13% for 2 h and 21% for 24 h for chemically treated fibers, and 20% for 2 h and 35% for 24 h for panels whose fibers were not treated. The explanation for these behaviors is the same—polymeric resins are in general hydrophobic and tend to not absorb water. Chamotte is hydrophilic, which means that they have affinity with water. If the amount of this particulate increases, we will have more water in the system.

Additionally, for thickness swelling, more specifically for the 24-h test, the ABNT 14810-1 [30] and EN 312 [34] standard establish acceptable variations for each type of application of the panel produced. Panels manufactured with 15% resin were classified as P7, the most rigid grade. Panels manufactured with 10% resin as P5 for T1 and T2 formulations, as P3 for T3 formulation, and T4 formulation cannot be classified.

When comparing the results of the present study with those previously cited, the similarity of results is noted, mainly for panels with 0%, 5%, and 10% of chamotte incorporated, for periods of 2 and 24 h.

Table 5 presents the Pearson correlation factors for the physical properties studied. Note that density has a moderate correlation only with moisture content and is weak or insignificant with other properties. Moisture content, water absorption at 2 and 24 h, and thickness swelling at 2 and 24 h are strongly correlated.

Table 6 shows the apparent density values for panels manufactured by hot pressing and determined by X-Ray densitometry and by gravimetric method, presenting a significant variation between treatments according to Tukey method, with a significance of 0.05.

**Figure 5:**
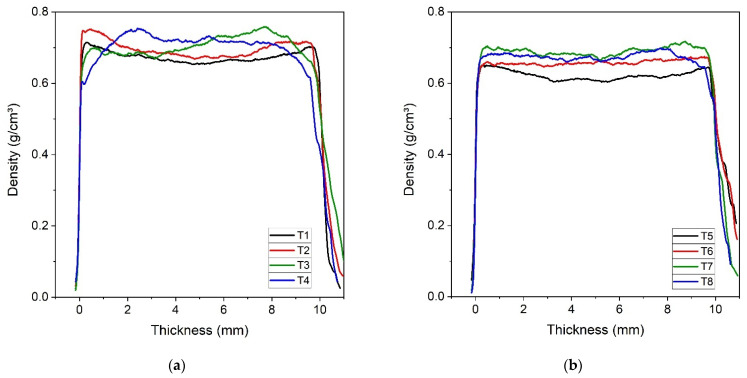
Density profiles obtained by X-ray densitometry. In (**a**) there are panels with 10% resin volumetric fraction and in (**b**) there are panels with 15% resin volumetric fraction.

Figure 5 shows the average apparent density profiles obtained in panels manufactured by hot pressing.

Through Figure 5 it is possible to observe that the incorporation of chamotte in the panels causes changes of density profile, in comparison what is usually expected format for a panel manufactured by hot pressing. Higher density values on the upper and lower faces, and lower on the center, are characteristics of panels manufactured by hot pressing. These characteristics affect the physical and mechanical properties, as mentioned by Lopez et al. [35], Martins et al. [36], and Wong et al. [37]. This behavior reveals that there is no homogeneity in the mixture, and then the chamotte was aggregated in some areas, and therefore decanted in some places. When we are using hot pressing, initially the resin viscosity drops low, and chamotte that is heavier than the other particulates tends to decant.

Table 7 presents the Pearson correlations for the results obtained in the X-Ray densitometry test for panels manufactured by hot pressing.

It is possible to observe that the average apparent density has a weak correlation with the maximum density, a strong correlation with the minimum density, and a very strong correlation with the gravimetric density according to Pearson’s correlation factor. This result of very high correlation between the average apparent density and the gravimetric density indicates that, for this type of composite panel, the X-ray densitometry is a reliable method.

**Figure 6:**
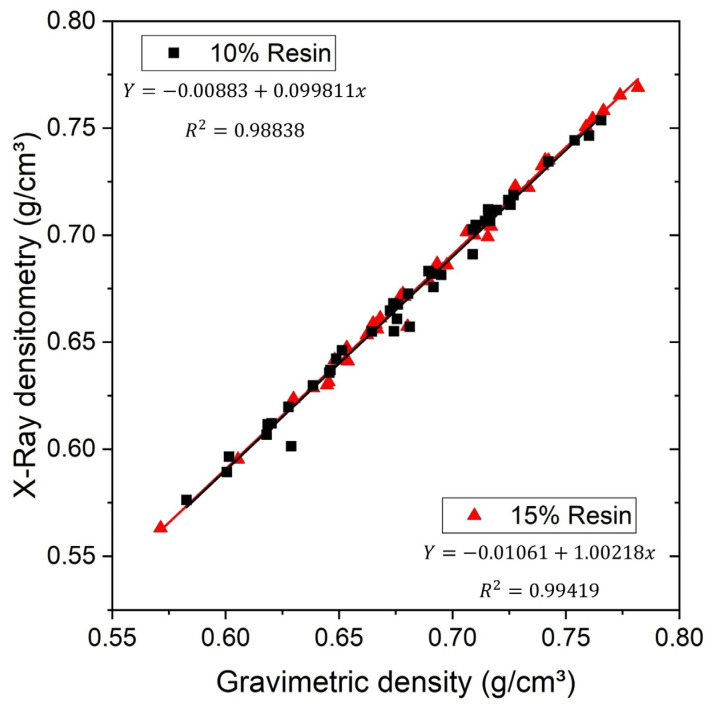
Correlation between densities obtained by X-ray and gravimetry.

Figure 6 shows a graph correlating the average apparent density obtained by X-ray densitometry and the gravimetric density obtained by the direct measurement method for resin volumetric fraction.

### Scanning Electron Microscopy

**Figure 7:**
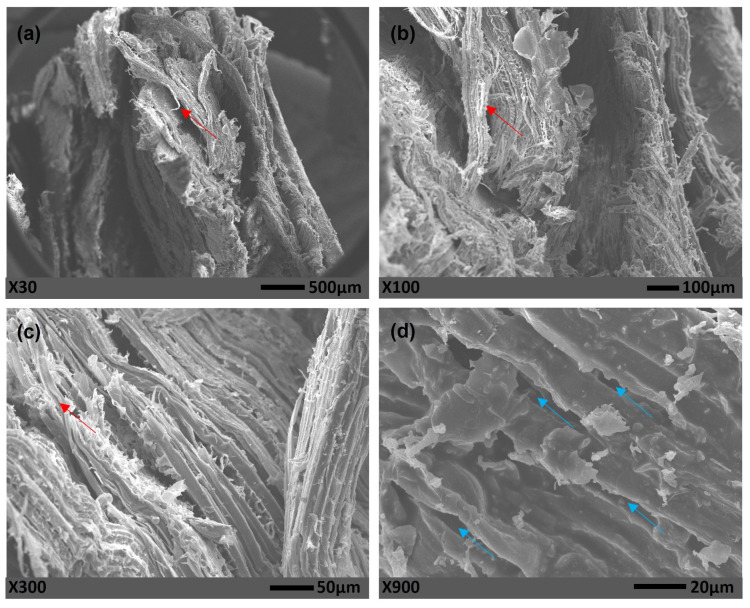
SEM of composites manufactured by hot pressing, without chamotte, (**a**–**c**) showing with different magnifications the fibers pulled out and (**d**) with high magnification the voids around the fibers.

In Figure 7, the images obtained by Scanning Electron Microscopy from composite panels manufactured in hot pressing conditions without the addition of chamotte in the formulation are presented.

Through Figure 7, it was not possible to notice the presence of bubbles in the polyurethane matrix, in addition to the fibers defibrillations and pulled out of matrix (red arrows), voids (blue arrows) around the eucalyptus fibers. No bubbles were observed in the matrix, which could be due to its low volume fraction in the composite panel.

It was observed in Figure 7a–c that there is a small amount of polyurethane resin, making it difficult to show it. This corroborates the low physical properties results obtained by hot-manufactured panels when compared to cold-manufactured ones [12].

**Figure 8:**
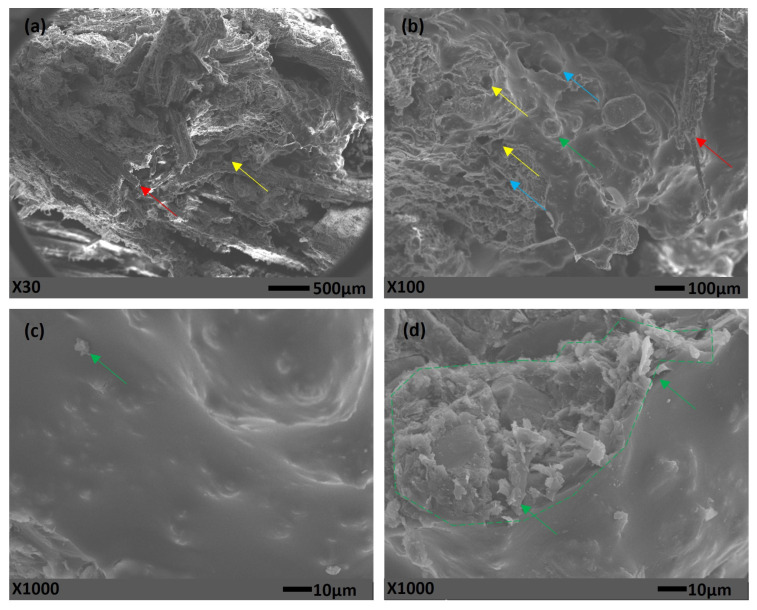
SEM composites manufactured by hot pressing, with chamotte.

Figure 8 presents the SEM images for the composite panels manufactured by hot pressing, with the addition of chamotte in its formulation.

It is possible to observe through Figure 8 that, with addition of chamotte (green arrows) in the formulation of hot-manufactured composite panels, the polyurethane matrix acquired a granular texture. In cold-manufactured panels [12], this granular texture is related to chamotte particles into the matrix, so the particles below 10µm tend to be embedded by the polymer matrix, probably because of low superficial tension with polyurethane resin.

This was different from the cold-manufactured composite panels. In these, the chamotte was homogenized after mixing the resin with the eucalyptus sawdust. This did not result in good homogenization, as can be seen in Figure 8d, where an agglomerate of chamotte particles is shown (green dashed outline) on the polyurethane matrix.

## 4. Conclusions

The results obtained demonstrate potential in the manufacturing of panels by hot pressing using eucalyptus sawdust waste, chamotte, and polyurethane resin based on castor oil.

When analyzing the physical properties surveyed, it was noted that there was a great influence of the volumetric fraction of resin, and the best results were found for 15% of resin. Chamotte also proved to be quite influential in the results, where the increase in the volumetric fraction of this residue incorporated into the panel caused an increase of investigated properties.

Regarding the parameters that classify the panels, it was possible to notice that most of the proposed formulations meet the minimum required, especially for thickness swelling, and all formulations with 15% resin met the most demanding requirement—being classified as P7 structural panels for use in severe load conditions in humid environments.

It is recommended to investigate new methodologies for homogenizing the resin in eucalyptus sawdust, in addition to carrying out new tests with other formulations and variations in temperature and pressing time.

## Figures and Tables

**Table 1:** Physical and chemical properties of individual components and the two-component polyurethane resin.

Property	Component A	Component B	Resin
Color	Brown	Amber	Amber
Consistency	Liquid	Liquid	Fluid
Density	1.22 g/cm^3^	0.96 g/cm^3^	1.05 g/cm^3^
Boiling point	330 °C	270–290 °C	-
Flash point	204 °C	280 °C	-
Solubility in water	Insoluble	Insoluble	-
Viscosity	170–250 mPa.s	-	-
Release of toxic elements	-	-	Free
Touch dry	-	-	60–90 min
Minimum mold removal time	-	-	6 h
Curing time and Handling	-	-	24 h
Heat resistance	-	-	Mass loss only after 210 °C

**Table 2:** Experimental formulations to produce panels by hot pressing.

Treatments	Resin (vol.%)	Chamotte (vol.%)	Eucalyptus Sawdust (vol.%)
T1	10	0	90
T2		5	85
T3		10	80
T4		15	75
T5	15	0	85
T6		5	80
T7		10	75
T8		15	70

**Table 3:** Results of physical properties analyzed for panels with 10 vol.% of resin.

Treatments	Density	Moisture Content	Water Absorption		Thickness Swelling	
	(g/cm^3^)	(%)	2 h	24 h	2 h	24 h
T1	0.653 ± 0.042 a	11.824 ± 1.004 a	11.501 ± 3.008 a	30.475 ± 7.197 a	3.261 ± 1.450 a	10.504 ± 4.870 a
T2	0.674 ± 0.045 a	12.369 ± 1.089 a	13.375 ± 5.131 a	43.839 ± 12.193 b	3.524 ± 0.892 a	11.441 ± 2.267 a
T3	0.695 ± 0.048 a	15.777 ± 1.661 b	23.696 ± 8.000 b	48.889 ± 5.905 bc	6.000 ± 2.475 b	17.619 ± 5.394 ab
T4	0.698 ± 0.041 a	17.978 ± 1.332 c	29.271 ± 7.653 b	57.679 ± 8.561 c	6.293 ± 1.083 b	20.626 ± 10.419 b
*p-value*	0.102142	5.701 × 10^−13^	2.476 × 10^−7^	5.571 × 10^−7^	6.0868 × 10^−5^	0.0026298

Means followed by the same letter, in the same column, do not differ by Tukey’s test at a 5% significance level.

**Table 4:** Results of physical properties analyzed for panels with 15 vol.% of resin.

Treatments	Density	Moisture Content	Water Absorption		Thickness Swelling	
	(g/cm^3^)	(%)	2 h	24 h	2 h	24 h
T5	0.672 ± 0.050 a	8.371 ± 1.010 a	5.761 ± 1.529 a	14.020 ± 3.767 a	1.258 ± 1.013 a	4.577 ± 2.011 a
T6	0.685 ± 0.053 a	10.265 ± 0.646 b	7.931 ± 2.104 ab	19.209 ± 4.248 ab	2.091 ± 1.026 ab	5.553 ± 1.580 a
T7	0.696 ± 0.041 a	11.293 ± 0.745 c	8.678 ± 3.027 b	21.123 ± 6.526 b	2.143 ± 0.738 ab	6.084 ± 3.216 a
T8	0.708 ± 0.043 a	15.489 ± 0.613 d	10.278 ± 2.367 b	24.081 ± 5.174 b	2.844 ± 0.996 b	7.138 ± 4.253 a
*p-value*	0.387058	1.523 × 10^−20^	0.001208	0.0007591	0.007524	0.28783

Means followed by the same letter, in the same column, do not differ by Tukey’s test at a 5% significance level.

**Table 5:** Pearson correlation for physical properties of panels manufactured by hot pressing.

	Density	Moisture Content	WA2h	WA24h	TS2h	TS24h
Density	1	0.5870729 *	0.333546	0.178303	0.297361	0.194244
Moisture Content	-	1	0.862667 *	0.804616 *	0.876088 *	0.825234 *
WA2h	-	-	1	0.943514 *	0.982379 *	0.985754 *
WA24h	-	-	-	1	0.954389 *	0.971426 *
TS2h	-	-	-	-	1	0.986341 *
TS24h	-	-	-	-	-	1

* Significant correlation (*p* ≤ 0.05). WA2h = 2 h water absorption; WA24h = 24-h water absorption; TS2h = thickness swelling at 2 h; TS24h = 24-h thickness swelling.

**Table 6:** Apparent densities of panels by X-Ray densitometry.

Treatments	Panels Apparent Density (g/cm^3^) by X-ray Densitometry			Average Gravimetry (g/cm^3^)
	Average	Maximum	Minimum	
T1	0.645 ± 0.043	0.708 ± 0.049	0.417 ± 0.092	0.653 ± 0.042
T2	0.663 ± 0.047	0.741 ± 0.043	0.438 ± 0.119	0.674 ± 0.045
T3	0.685 ± 0.048	0.766 ± 0.071	0.502 ± 0.066	0.695 ± 0.048
T4	0.687 ± 0.042	0.776 ± 0.052	0.493 ± 0.082	0.698 ± 0.041
T5	0.663 ± 0.048	0.657 ± 0.058	0.378 ± 0.069	0.672 ± 0.050
T6	0.676 ± 0.054	0.701 ± 0.077	0.460 ± 0.056	0.685 ± 0.053
T7	0.687 ± 0.042	0.739 ± 0.091	0.492 ± 0.124	0.696 ± 0.041
T8	0.699 ± 0.044	0.719 ± 0.045	0.479 ± 0.069	0.708 ± 0.043

**Table 7:** Pearson correlations of results obtained by X-ray densitometry of panels manufactured by hot pressing.

	Average Apparent Density	Maximum Density	Minimum Density	Gravimetry Density
Average Apparent Density	1	0.457009878	0.795819531 *	0.997798 *
Maximum Density	-	1	0.832633845 *	0.484177
Minimum Density	-	-	1	0.796531 *
Gravimetry Density	-	-	-	1

* Significant correlation (*p* ≤ 0.05).

## References

[1] FAOSTAT Particleboard Production Quantity in World and Brazil. http://www.fao.org/faostat/en/#data/FO/visualize.

[2] Auriga R., Auriga A., Borysiuk P., Wilkowski J., Fornalczyk O., Ochmian I. (2022). Lignocellulosic Biomass from Grapevines as Raw Material for Particleboard Production. Polymers.

[3] Lee S.H., Lum W.C., Boon J.G., Kristak L., Antov P., Pędzik M., Rogoziński T., Taghiyari H.R., Lubis M.A.R., Fatriasari W. (2022). Particleboard from agricultural biomass and recycled wood waste: A review. JMRT.

[4] Dionizio A.F., Vale A.T., Moreira A.C.O., Galvão L.G.O., Chaves B.S., Costa M.A. (2019). Adding value to agro-industrial waste for energy purposes. Rev. De CiÊNcias AgrÁRias.

[5] Pędzik M., Janiszewska D., Rogoziński T. (2021). Alternative lignocellulosic raw materials in particleboard production: A review. Ind. Crops Prod..

[6] Klímek P., Wimmer R., Meinlschmidt P., Kúdela J. (2018). Utilizing Miscanthus stalks as raw material for particleboards. Ind. Crops Prod..

[7] Paula L.E.R., Trugilho P.F., Napoli A., Bianchi M.L. (2011). Characterization of residues from plant biomass for use in energy generation. Cerne.

[8] Oliveira J.N., Lopes F.P.D., Simonassi N.T., Souza D., Monteiro S.N., Vieira C.M.F. (2023). Evaluation of the physical properties of composite panels with eucalyptus sawdust waste and castor oil-based polyurethane. JMRT.

[9] Marchi B.Z., Oliveira M.S., Bezerra W.B.A., Sousa T.G., Candido V.S., Silva A.C.R., Monteiro S.N. (2022). Ubim Fiber (Geonoma baculífera): A Less Known Brazilian Amazon Natural Fiber for Engineering Applications. Sustainability.

[10] Ribeiro M.M., Pinheiro M.A., Rodrigues J.S., Ramos R.P.B., Corrêa A.C., Monteiro S.N., Silva A.C.R., Candido V.S. (2022). Comparison of Young’s Modulus of Continuous and Aligned Lignocellulosic Jute and Mallow Fibers Reinforced Polyester Composites Determined Both Experimentally and from Theoretical Prediction Models. Polymers.

[11] Oliveira Filho E.G., Luz F.S., Fujiyama R.T., Silva A.C.R., Candido V.S., Monteiro S.N. (2020). Effect of Chemical Treatment and Length of Raffia Fiber (Raphia vinifera) on Mechanical Stiffening of Polyester Composites. Polymers.

[12] Ribeiro D.P., Vilela A.P., Silva D.W., Napoli A., Mendes R.F. (2019). Effect of Heat Treatment on the Properties of Sugarcane Bagasse Medium Density Particleboard (MDP) Panels. Waste Biomass Valorization.

[13] Vieira C.M.F., Holanda J.N.F., Pinatti D.G. (2000). Caracterização de massa cerâmica vermelha utilizada na fabricação de tijolos na região de Campos dos Goytacazes—RJ. Cerâmica.

[14] Vieira C.M.F., Souza E.T.A., Monteiro S.N. (2004). Efeito da incorporação de chamote no processamento e microestrutura de cerâmica vermelha. Cerâmica.

[15] Silva D.W., Farrapo C.L., Ribeiro D.P., Mendes R.F., Mendes L.M., Scolforo J.R.S. (2015). MDP com partículas de eucalipto e palha de milho. Sci. For..

[16] Valle A.C.M., Ferreira B.S., Prates G.A., Goveia D., Campos C.I. (2020). Physical and mechanical properties of particleboard from eucalyptus grandis produced by urea formaldehyde resin with SiO_2_ nanoparticles. Eng. AgrÍCola.

[17] Morais W.W.C., Haselein C.R., Susin F., Vivian M.A., Souza J.T. (2018). Uso de Bambusa tuldoides e Eucalyptus grandis para confecção de painéis aglomerados. CiÊNcia Florest..

[18] Mendes R.F., Mendes L.M., Guimarães Júnior J.B., Mori F.A., César A.A.S. (2010). Efeito da incorporação de casca de café nas propriedades físico-mecânicas de painéis aglomerados de Eucalyptus urophylla S.T. Blake. CiÊNcia E Agrotecnol..

[19] Silva V.U., Nascimento M.F., Oliveira P.R., Panzera T.H., Rezende M.O., Silva D.A.L., Aquino V.B.M., Lahr F.A.R., Christoforo A.L. (2021). Circular vs. linear economy of building materials: A case study for particleboards made of recycled wood and biopolymer vs. conventional particleboards. Constr. Build. Mater..

[20] Ghani A., Ashaari Z., Bawon P., Lee S.H. (2018). Reducing formaldehyde emission of urea formaldehyde-bonded particleboard by addition of amines as formaldehyde scavenger. Build. Environ..

[21] Wechsler-Pizarro A., Nðñez-Decap M. (2022). Preliminary study of particleboards manufactured with pine cones and Eucalyptus globulus capsules with a bio-based polyurethane adhesive. Polym. Compos..

[22] Bispo R.A., Trevisan M.F., Silva S.A.M., Aquino V.B.M., Saraiva R.L.P., Arroyo F.N., Molina J.C., Chahud E., Branco L.A.M.N., Panzera T.H. (2022). Production and evaluation of particleboards made of coconut fibers, pine, and eucalyptus using bicomponent polyurethane-castor oil resin. Bioresources.

[23] Sugahara E.S., Silva S.A.M., Buzo A.L.S.C., Campos C.I., Morales E.A.M., Ferreira B.S., Azambuja M.A., Lahr F.A.R., Christoforo A.L. (2019). High-density particleboard made from agro-industrial waste and different adhesives. Bioresources.

[24] (1993). Wood-Based Panels—Determination of Density.

[25] (1993). Wood-Based Panels—Determination of Moisture Content.

[26] (1993). Wood-Based Panels—Determination of Swelling in Thickness after Immersion in Water.

[27] (2018). Painéis de Partículas de Média Densidade—Parte 2: Requisitos e Métodos de Ensaio.

[28] Buzo A.L.S.C., Sugahara E.S., Silva S.A.M., Morales E.A.M., Azambuja M.A. (2019). Painéis de pínus e bagaço de cana empregando-se dois adesivos para uso na construção civil. Ambiente ConstruÍDo.

[29] Santos W.L.F., Silva A.J.P., Cabral A.A., Mercury J.M.R. (2014). Particleboard manufactured from Tauari (Couratari oblongifolia) wood waste using castor oil based polyurethane resin. Mater. Res..

[30] (2013). Painéis de Partículas de Média Densidade—Parte 1: Terminologia.

[31] Wechsler A., Zaharia M., Crosky A., Jones H., Ramírez M., Ballerini A., Nuñez M., Sahajwalla V. (2013). Macadamia (Macadamia integrifolia) shell and castor (Rícinos communis) oil based sustainable particleboard: A comparison of its properties with conventional wood based particleboard. Mater. Des..

[32] Bertolini M.S., Morais C.A.G., Lahr F.A.R., Freire R.T.S., Panzera T.H., Christoforo A.L. (2019). Particleboards from CCB-Treated Pinus sp. Wastes and Castor Oil Resin: Morphology analyses and physical-mechanical properties. J. Mater. Civ. Eng..

[33] Mesquita A.L., Barrero N.G., Fiorelli J., Christoforo A.L., Faria L.J.G., Lahr F.A.R. (2018). Eco-particleboard manufactured from chemically treated fibrous vascular tissue of acai (*Euterpe oleracea Mart*) Fruit: A new alternative for the particleboard industry with its potential application in civil construction and furniture. Ind. Crops Prod..

[34] (2010). Particleboards—Specifications.

[35] Lopez Y.M., Gonçalves F.G., Paes J.B., Gustave D., Segundinho P.G.A., Latorraca J.V.F., Nantet A.C.T., Suuchi M.A. (2021). Relationship between internal bond properties and x-ray densitometry of wood plastic composite. Compos. Part B Eng..

[36] Martins R.S.F., Gonçalves F.G., Segundinho P.G.A., Lelis R.C.C., Paes J.B., Lopez Y.M., Chaves I.L.S., Oliveira R.G.E. (2021). Investigation of agro-industrial lignocellulosic wastes in fabrication of particleboard for construction use. J. Build. Eng..

[37] Wong M.C., Hendrikse S.I.S., Sherrell P.C., Ellis A.V. (2020). Grapevine waste in sustainable hybrid particleboard production. Waste Manag..

